# Actinomycosis of the Middle Ear Mimicking Cholesteatoma: A Case Report and Literature Review

**DOI:** 10.7759/cureus.55014

**Published:** 2024-02-27

**Authors:** Shagoon Modi, Eleftheria Kiverniti

**Affiliations:** 1 Surgery, Princess Alexandra Hospital, Harlow, GBR; 2 Otolaryngology, Princess Alexandra Hospital, Harlow, GBR

**Keywords:** cholesteatoma surgery, actinomycosis cervicofacial, actinomyces species, middle ear infection, actinomyces infections

## Abstract

Actinomycosis is a rare infection of the middle ear. Actinomyces is an anaerobic, filamentous bacterium causing granuloma formation and suppurative infection. We present a young male with a nine-month history of unilateral, yellow-coloured otorrhoea and hearing loss. Swabs showed no growth, with the infection not responding to oral or topical antibiotics. Computed tomography of temporal bones was consistent with cholesteatoma and ossicular disruption. Surgical exploration revealed a yellow, cystic mass within the middle ear. Cortical mastoidectomy and washout were performed. Histological diagnosis confirmed Actinomyces clusters with positive gram stain. Actinomycosis of the middle ear typically presents as chronic otitis media. It likely reaches the middle ear via the eustachian tube. It is often misdiagnosed due to culture insensitivity; however, clinical suspicion can aid labs in providing an optimum culture environment. Tympanomastoidectomy allows for histopathological diagnosis. Surgical resection should be followed by a prolonged course of antibiotics.

## Introduction

Actinomycosis is a rare infection of the middle ear. It is a saprophytic infection caused by Actinomyces. Actinomyces is a gram-positive, anaerobic, non-acid-fast, filamentous bacterium [1,2]. They are part of the normal flora of the oropharynx, digestive, and genital tracts [3]. The infection is characterised by granuloma formation, suppurative inflammation, and discharge of sulphur granules. There are less than 50 documented cases worldwide of cervicofacial actinomycosis, with the most recent case reported in the UK in 2013 - the first in 60 years [4,5]. Beck was the first to describe an otologic case of actinomycosis in 1906. Middle-ear actinomycosis is most common in children to middle-aged adults; over 50% of cases occur in those under the age of 15 years [6].

It is believed there are a few different routes for contamination of the middle ear. Firstly, via the nasopharynx through the eustachian tube into the middle ear. This may be due to mucosal injury following trauma, such as tears to the mucosa, dental extraction, and dental caries [6,7]. This route is considered the most likely. In addition, children may be more susceptible to middle ear actinomycosis because they are prone to ascending infections via the eustachian tube [6]. Second, the direct route via the external auditory canal, secondary to external trauma and perforation of the tympanic membrane. There is a possibility of inoculation following myringotomy too [8]. Lastly, the haematogenous route; actinomycosis spread via the bloodstream is less likely and only possible in major infections [7].

Once in the middle ear, Actinomyces spread via contiguous and direct extension regardless of anatomical barriers [9]. These organisms form an inflammatory granuloma with a fibrous pseudo-capsule, appearing as yellow granules macroscopically [8]. Middle ear actinomycosis is characterised by abscesses, tissue fibrosis, and draining sinuses [1].

Before the antibiotic era, actinomycosis was seldom detected prior to autopsy - most of these infections were fatal due to their intracranial involvement [1]. Some of the intracranial complications include petrous apicitis, subdural abscess, and sinus thrombosis [7]. Miller et al. [2] reported a case in 2014 of an otogenic brain abscess, which reminds us that despite a reduced incidence of actinomycosis, intracranial complications continue to occur. Other complications are facial nerve palsy, osteomyelitis, and labyrinthine fistula [10,11].

Here, we present a case that was seen at a district general hospital in England.

This article was previously presented as a poster at three conferences: the Association of Surgeons in Training Annual Conference 2023 on 4th March 2023, the Association of Otolaryngologists in Training Annual National Conference 2022 on 17th June 2022, and the A to Z in Head and Neck Surgery Summer Conference 2022 on 18th June 2022.

## Case presentation

Mr. O, a 35-year-old male, presented to the ENT clinic with a nine-month history of right-sided otorrhoea. He had been referred by his general practitioner (GP) who had prescribed oral antibiotics and Otomize ear spray with little success. Ear swabs taken by the GP showed no significant growth. He had a clear and yellow-coloured discharge, which he initially noticed on his pillow in the morning. He did not find the discharge to be foul-smelling and denied any itching, vertigo, or symptoms of the left ear. There was occasional, sharp pain behind the affected ear. He described a new crackling sound in his ear when swallowing and a long-standing whooshing noise that had been present for years. He also complained of some right-sided hearing loss.

Mr. O underwent grommet insertion in childhood and once in adult life. His past medical history was not significant apart from phenylketonuria.

On examination in the clinic, the right side showed otorrhoea. When this was microsuctioned, it revealed an area of granulation tissue on the pars tensa and a bulging tympanic membrane; this was attributed to the previous grommets, as one could no longer be visualised. Apart from microsuction, this was treated with a course of Sofradex drops and he was advised strict caution with water in the right ear. At his two-month follow-up, Mr. O showed no improvement in symptoms. His ear was microsuctioned once again and filled with a mixture of a steroid and antibiotic cream. Computed tomography (CT) scan was arranged to investigate any communication with the middle ear.

**Figure 1:**
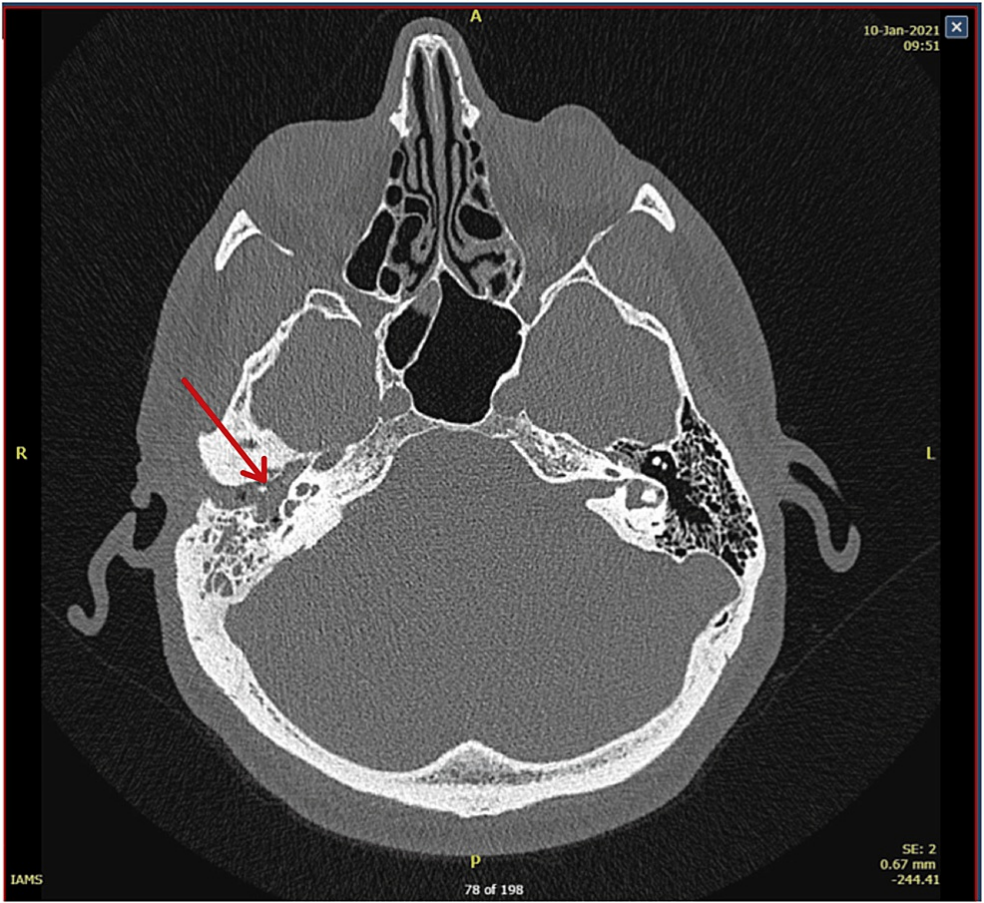
CT of the temporal bones. A CT scan of the temporal bones revealed opacification in the right middle ear, highlighted by an arrow, suggesting erosion of the malleus and incus.

CT of the temporal bones showed opacification of the right middle ear with erosion of the malleus and incus, as seen in Figure 1. Appearances were noted to be suspicious for cholesteatoma. Apart from that, there was right-sided otitis externa and incidental left maxillary sinusitis. Due to the consistent findings of cholesteatoma and ossicular disruption in the CT scan, and considering his conductive hearing loss, Mr. O was scheduled for an ossiculoplasty and mastoidectomy.

**Figure 2:**
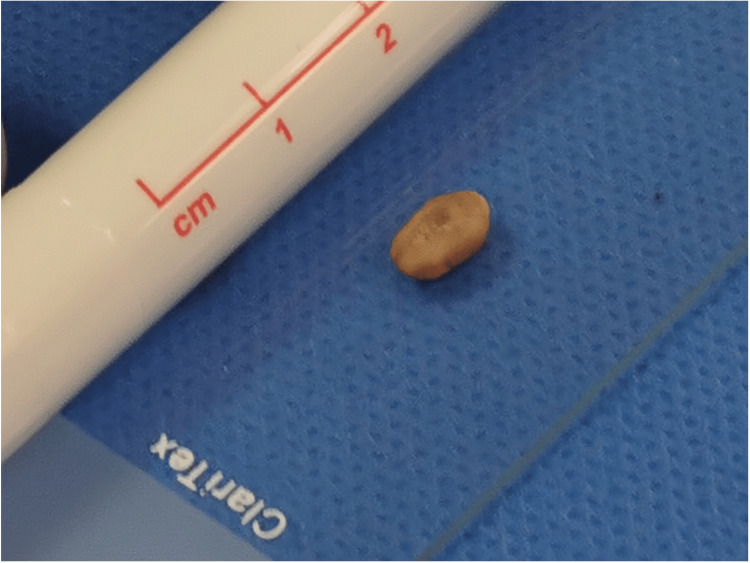
Specimen retrieved from the middle ear. An 8 x 8 x 3 mm mass was found in the middle ear, with yellow discolouration and relatively firm consistency.

During the surgical exploration of his right ear, the tympanic membrane was found to be bulging and featureless, without perforations or retractions. A cystic mass was found to be filling the middle ear with a strange structure contained inside. The structure had yellow discolouration and was relatively hard (Figure 2). This was sent off for histopathology. The mass was surrounded by granulation tissue and a substantial amount of pus-like discharge. No true cholesteatoma was found. Ossicles were present, intact and connected despite a CT scan suggesting otherwise. Cortical mastoidectomy and middle ear washout were performed.

Histology reported two separate fragments measuring 8 x 8 x 3 mm in aggregate. The section showed clusters of Actinomyces organisms, which stained positive with gram stain.

Following microbiology advice, he was prescribed a three-month course of oral amoxicillin. A follow-up appointment confirmed the resolution of symptoms, and an outpatient hearing test was scheduled for the upcoming months.

## Discussion

Clinical features

Actinomycosis of the middle ear typically presents with recurrent otorrhoea similar to chronic suppurative otitis media [4]. It often has a prolonged course, which recurs intermittently and is resistant to numerous antibiotics [10]. A low-grade fever may be found in 50% of cases, but pain is an uncommon symptom [11]. Hearing impairment is a documented feature in several cases [6]. Clinical examination typically reveals a perforated or bulging tympanic membrane that may be pink or red [12]. It can often mimic malignancy and be misdiagnosed as such [6].

Diagnosis

As otoscopy is often inconclusive, clinicians may opt for CT imaging; this usually shows a thickened eardrum and soft tissue within the middle ear [7]. Thus, imaging is not useful apart from illustrating the extent of the infection [10]. Tympanomastoidectomy is almost essential as it allows for debulking and histopathological diagnosis of actinomycosis; to date, it is the most common means of doing so [4].

Swabs taken from the middle ear do not help with isolation and identification of the causative organism, with cultures being falsely negative in over 70% of cases [4,11]. Instead, other organisms that are co-existent might be detected [6]. Cultures are often negative due to inadequate culture conditions; Actinomyces require rapid transport to a lab, with anaerobic processing and growth conditions, along with a culture period of at least five days and up to 20 days [3]. For these reasons, informing the lab of a suspected Actinomyces infection may be useful to provide an optimum culture environment.

Surgically resected biopsy or pus drained from the collection are the most suitable specimens for diagnosis [3]. Microscopic findings include yellow, glue-like masses - often called sulphur granules - and filamentous, gram-positive organisms [3,13]. These sulphur granules are constituted by a dense mass of Actinomyces surrounded by a complex of polysaccharides, calcium, and proteins [8]. The granules are contained within granulomatous inflammatory tissue [10]. Shelton and Brackmann [12] suggest that two of the following criteria must be present for diagnosis of actinomycosis: positive culture, sulphur granules, or biopsy showing the organism.

Multiple novel diagnostic techniques are currently being trialled, such as 16S ribosomal ribonucleic acid (rRNA) gene sequence analysis, mass spectrometry, and polymerase chain reaction [5,14]. These techniques have superior sensitivity and specificity compared to more traditional existing methods [9]. However, they are not used routinely due to their lack of availability, lack of experience in using them, difficulty obtaining bacterial DNA from clinical specimens, and susceptibility to contaminants [9].

Differentials

In chronic otorrhoea with negative cultures, the differential diagnosis includes malignancy, cholesteatoma, tuberculosis, and nocardiosis [7,15]. Tuberculosis may present with chronic otorrhoea and hearing loss, similar to actinomycosis; however, it is sometimes associated with facial paralysis [10]. Sulphur granules are almost pathognomonic for Actinomyces, but Nocardia also forms them [6]. Nocardiosis is also a granulomatous and suppurative infection, but it usually causes a more acute infection than Actinomyces [9]. Nocardia can be differentiated from Actinomyces as it is positive on acid-fast staining [6].

Management

Middle ear actinomycosis is best treated with surgical debridement by tympanomastoidectomy. Surgery allows for direct clearance of the infected tissue in addition to aerating the site - this is thought to be crucial as Actinomyces are anaerobic organisms [5,12]. Debridement is often necessary as Actinomyces can reside within poorly vascularised tissue where antibiotics may not reach their therapeutic concentrations [16].

Surgical intervention must be followed by a prolonged course of antibiotics to achieve long-term disease remission [5]. Most authors describe successful disease remission with oral penicillin or amoxicillin for three to six months [3,8,13]. A prolonged course of antibiotics, at a high dose facilitates optimal antibiotic penetration in abscesses and infected tissues [3]. Penicillin-allergic patients can be treated with tetracycline, erythromycin, clindamycin, and chloramphenicol [11]. Topical treatment is not recommended as Actinomyces is resistant to ciprofloxacin, and other topical antibiotics - when used for an extended period - may cause ototoxicity [4].

In certain cases, non-surgical treatment may be possible; this is an option for non-severe infections, where there is good ventilation and drainage [10]. In these cases, a more extended antibiotic course of six to 12 months is considered appropriate [4]. This is made possible through early diagnosis of Actinomyces using tissue biopsy or culture [16]. Ideally, a greater awareness and understanding of the clinical presentation of Actinomyces will lead to earlier detection and therefore, better clinical response to non-surgical treatment [16].

A long-term follow-up is recommended after treatment of actinomycosis. Normal tympanic membrane and hearing are evidence of recovery [7]. However, follow-up with CT and MRI can rule out the presence of bony erosion and granulation tissue and indicate the degree of middle ear aeration [4].

## Conclusions

In conclusion, actinomycosis of the middle ear, although rare, is a serious infection often misdiagnosed due to culture insensitivity. It commonly presents as chronic, recurrent otitis media. Actinomyces likely reach the middle ear via the eustachian tube following dental work or direct trauma to the mucosa. Once in the middle ear, it forms an inflammatory granuloma, which appears as yellow, sulphur granules. The above case presented as a cholesteatoma clinically and radiologically, which delayed appropriate diagnosis and management. It also affected the patient's hearing.

Tympanomastoidectomy or pus drained from the infection site allows for histopathological diagnosis of the causative organism. Although cultures are often negative, clinical suspicion for Actinomyces can aid laboratories in providing an optimum culture environment. There are various options for newer diagnostic techniques, such as gene sequence analysis and mass spectrometry, but these are not used routinely. The recommended treatment for actinomycosis involves surgical resection of the infected tissue followed by a three to six-month course of antibiotics, typically using penicillin. Early diagnosis of Actinomyces may allow non-surgical treatment of the infection, as well as preventing intracranial complications. Long-term follow-up is recommended to ensure disease remission.
